# Fat biopsy from a pocket of cardiac implantable electronic device: An alternative diagnostic option for cardiac amyloidosis

**DOI:** 10.1016/j.hrcr.2022.05.008

**Published:** 2022-05-18

**Authors:** Ryo Takano, Nobuhiko Ueda, Atsushi Okada, Manabu Matsumoto, Yoshihiko Ikeda, Kinta Hatakeyama, Chisato Izumi, Kengo Kusano

**Affiliations:** ∗ Department of Cardiovascular Medicine, National Cerebral and Cardiovascular Center, Osaka, Japan; † Department of Pathology, National Cerebral and Cardiovascular Center, Osaka, Japan

**Keywords:** Atrioventricular block, Amyloid cardiomyopathy, Transthyretin, Conduction disease, Pacemaker, Cardiac implantable electronic device, Biopsy, Subcutaneous fat

**Key Teaching Points**

• As conduction disease is common in cardiac amyloidosis (CA), some cases with conduction disease should be assessed for latent CA.
• With the advent of novel disease-modifying therapeutic options, early accurate diagnosis of CA is becoming increasingly important.
• A fat biopsy from a device pocket could be an alternative option for histological confirmation and early diagnosis.

## Introduction

Transthyretin cardiac amyloidosis (TTR-CA) is a life-threatening disease, and advances in imaging techniques have allowed us to recognize that it has been underdiagnosed.^1^, ^2^, ^3^ Transthyretin (TTR) stabilizers and genetic silencers are novel disease-modifying therapeutic options for TTR amyloidosis, and the early and accurate diagnosis of TTR-CA is thus becoming increasingly important.

TTR-CA often presents as a conduction disorder together with atrial and ventricular arrhythmias.^4^ We report a case of syncope and advanced atrioventricular block (AVB) requiring pacemaker implantation, in which a fat biopsy from the pacemaker pocket led to a histological diagnosis of TTR-CA.

## Case report

**Figure 1:**
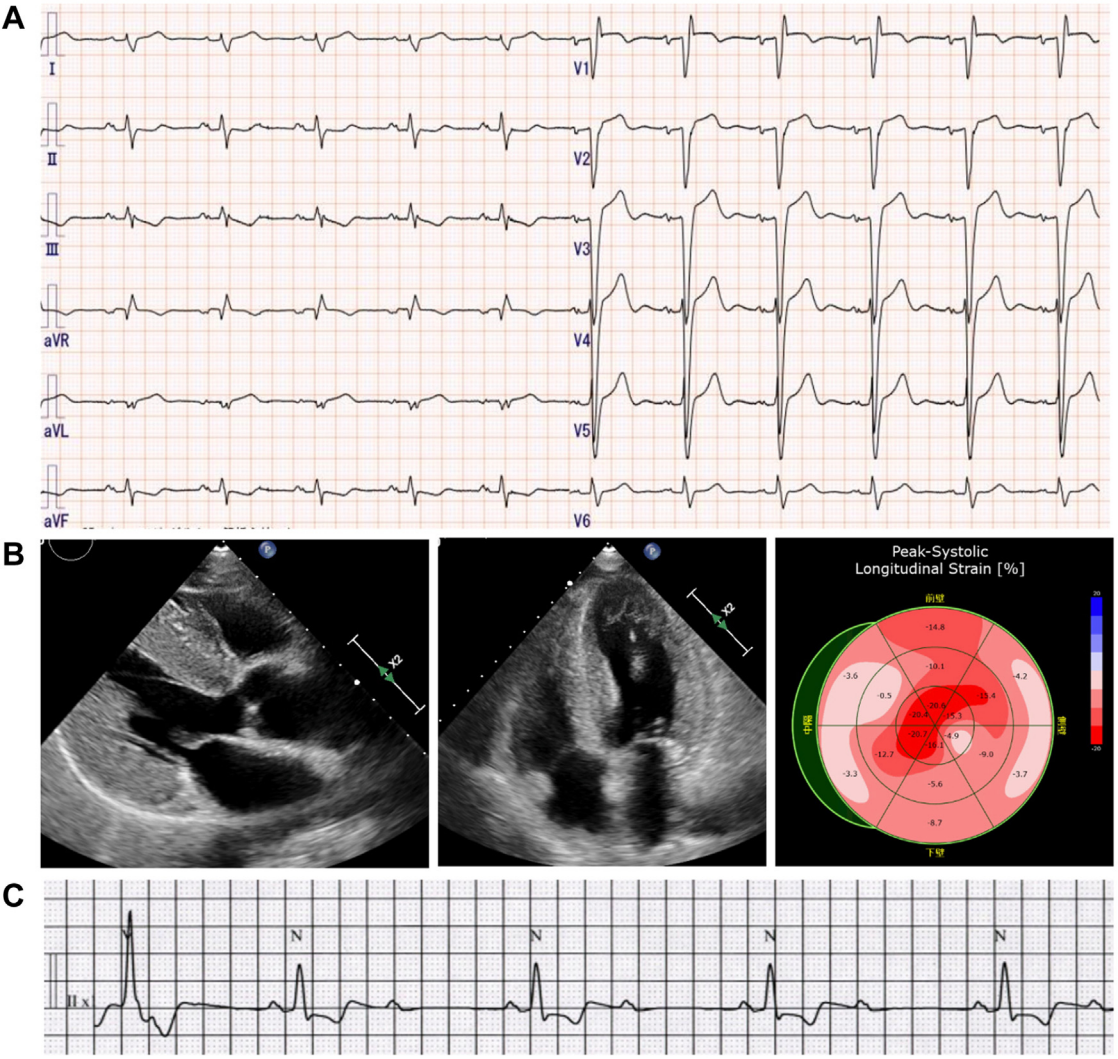
**A:** Electrocardiogram (ECG) showing sinus rhythm, intraventricular conduction delay with indeterminate axis, QS wave in leads V_1_–V_3_, and low R-wave amplitude in lead V_4_. **B:** Echocardiogram showing left ventricular hypertrophy and apical sparing pattern. **C:** Telemetry ECG showing an episode of intermittent 2:1 advanced atrioventricular block.

A 72-year-old Japanese man was referred to our emergency department for recurrent syncope. He had a past medical history of hypertension and lumbar spinal stenosis. He had no family history of cardiovascular disease or arrhythmias. Physical examination showed a normal jugular venous pulse, normal heart sounds with no murmur, clear lung fields, no lower-extremity edema, and no carpal tunnel syndrome, diarrhea, or polyneuropathy. Laboratory testing showed elevation of cardiac troponin T at 0.057 ng/mL (reference range: <0.014 ng/mL) and B-type natriuretic peptide at 121.8 pg/mL (reference range, <18.4 pg/mL). Electrocardiogram (ECG) on admission showed sinus rhythm, intraventricular conduction delay with indeterminate axis, QS wave in leads V_1_–V_3_, and low R-wave amplitude in lead V_4_ (Figure 1A). Chest radiograph showed mild cardiomegaly. Echocardiography showed left ventricular (LV) hypertrophy (interventricular septum/posterior wall thickness 13/14 mm), LV ejection fraction 62%, and LV global longitudinal strain exhibiting an apical sparing pattern (Figure 1B).

**Figure 2:**
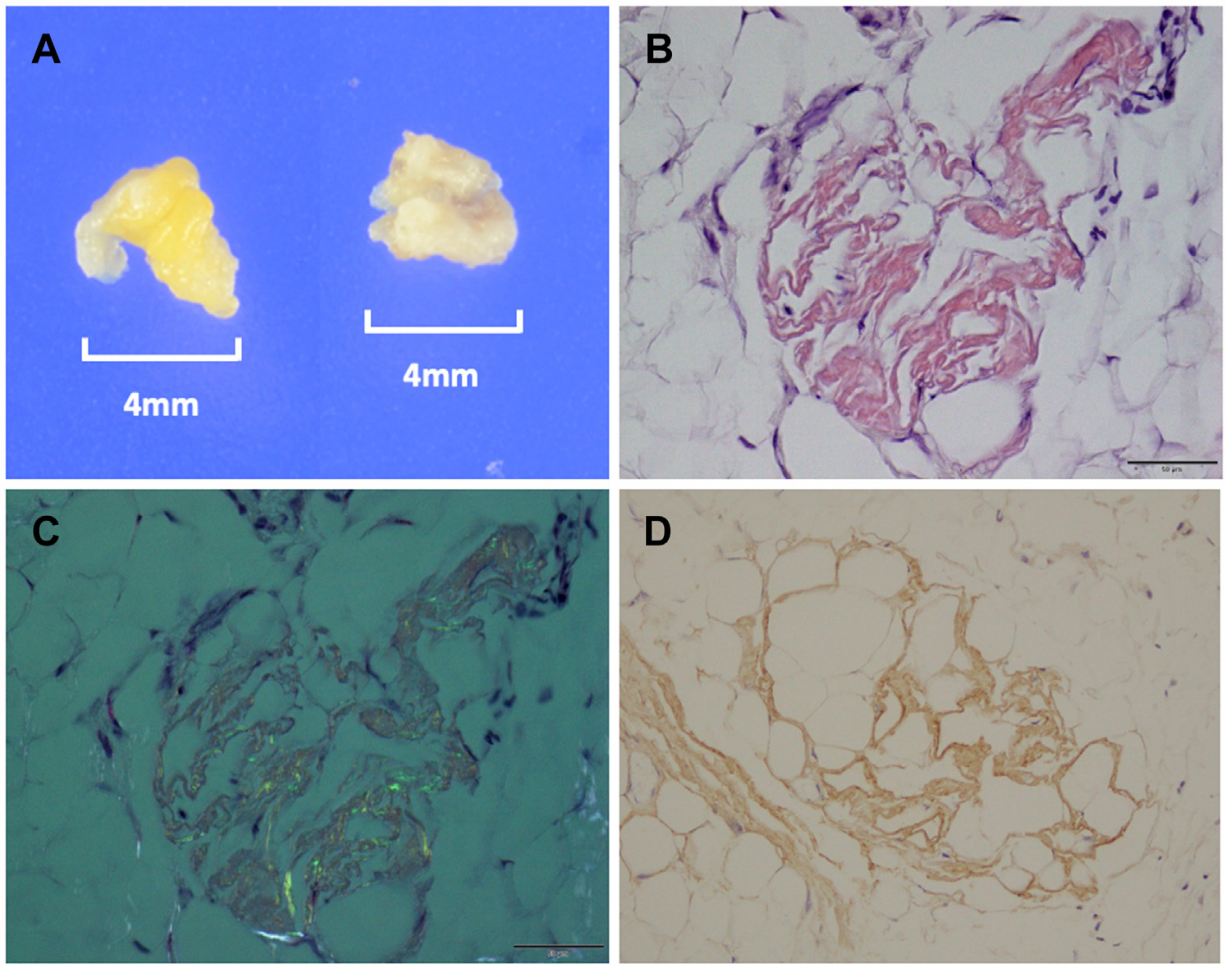
**A:** Two samples of 4-mm-sized subcutaneous fat from the pacemaker pocket. **B:** Congo red staining of fat tissue showing the salmon pink color. **C:** Congo red staining under polarized light showing the apple green birefringence. **D:** Immunohistochemical staining was positive for transthyretin.

After admission, an intermittent 2:1 advanced AVB was documented (Figure 1C). As the cause of syncope was considered to be bradycardic arrhythmia, we implanted a dual-chamber pacemaker (Azure; Medtronic Inc, Minneapolis, MN). The tined lead was positioned at the right atrial appendage, and the screw-in lead was positioned at the right ventricular septum with a C315 catheter delivery system. The pacing threshold and sensing amplitude were as follows: right atrial and right ventricle pacing threshold was 0.875 V / 0.4 ms and 0.375 V / 0.4 ms; sensing amplitude was 1.875 mV and 5.0 mV, respectively. As the previous findings (LV hypertrophy with apical sparing, increased troponin T, conduction disease) strongly suggested the possibility of cardiac amyloidosis (CA), we performed a fat tissue biopsy from the pacemaker pocket during the pacemaker implantation procedure for early diagnosis. Two samples of 4-mm-sized subcutaneous fat were obtained from differential points (Figure 2A). Both samples showed amyloid deposits by Congo red staining (Figure 2B) with apple green birefringence under polarized light (Figure 2C), and immunohistochemical staining was positive for TTR (Figure 2D).

Additional workup revealed that serum and urine protein electrophoresis with immunofixation showed no monoclonal proteins, and serum free light chains were within normal limits. ^99m^Technetium pyrophosphate scintigraphy showed grade 3 cardiac uptake with a heart/contralateral ratio of 2.03. These findings were compatible with TTR-CA. The patient had no heart failure symptoms and required no medications at the time of diagnosis; thus the patient is closely followed up at our heart failure clinic for development of heart failure symptoms and for future use of disease-modifying treatments including tafamidis for TTR-CA.

## Discussion

In this case, we confirmed intraventricular conduction delay with indeterminate axis on ECG and intermittent 2:1 advanced AVB on monitored ECG. Conduction disease is highly prevalent in CA, and atrioventricular conduction delay involving the His-Purkinje system appears to be more common than pure sinus node disease.^4^ In a report of 65 patients with TTR-CA, 43% of patients with wild-type TTR-CA and 36% of patients with variant TTR-CA had implanted pacemakers.^5^

Radionuclide bone scintigraphy with technetium-labeled bisphosphonates has been widely recognized as a useful test for diagnosing TTR-CA^6^; however, false-positives in amyloid light-chain amyloidosis, hypertrophic cardiomyopathy, and false-negatives have recently been reported in multiple reports.^7^, ^8^, ^9^, ^10^, ^11^ Therefore, histological confirmation of the diagnosis of amyloidosis remains essential in some cases.

Myocardial biopsy has high diagnostic accuracy (near 100%) for the diagnosis of CA^12^; however, it is also considered to be highly invasive and it includes risks for cardiac tamponade and complete AVB, and is often avoided in the elderly. Extracardiac biopsy including abdominal fat aspiration and skin biopsy can be performed less invasively; however, its sensitivity is inferior to myocardial biopsy.^12^ In a report of 600 patients with CA who underwent abdominal fat aspiration, the diagnostic accuracy of TTR-CA was only 15% in wild-type TTR-CA,^13^ and the positive rate of skin biopsy for wild-type TTR amyloidosis was 73%–79%.^14^^,^^15^ In addition, in skin biopsies, amyloid deposits are known to be observed mainly in the deep layer of subcutaneous fat tissue and to exhibit a patchy distribution; thus, failure to obtain a deep subcutaneous fat layer in skin biopsy could result in the lower positive rate.^14^ We believe that fat biopsy from a device pocket not only enables sample collection without additional invasion, but also allows the easy collection of samples from the deep subcutaneous fat layer, which could result in higher sensitivity. Further investigation is required on the diagnostic accuracy of fat biopsy from a device pocket.

## Conclusion

We report a case of CA that required device implantation and was histologically diagnosed by fat biopsy from a device pocket. In cases requiring cardiac implantable electronic device implantation with clinical findings suggesting CA, such as LV hypertrophy with apical sparing, increased troponin T, or conduction disease, fat biopsy from a device pocket could be an alternative option for histological diagnosis without additional invasiveness.
